# In Silico Evaluation of Cyanobacteria-Derived Bioactive Compounds as Potential Monoamine Oxidase-B Inhibitors for Parkinson’s Disease: Molecular Docking and Molecular Dynamics Simulation Study

**DOI:** 10.3390/ph19101569

**Published:** 2026-10-02

**Authors:** Sarah Khalid, Suhail Ahmad, Aman Chandra Kaushik, Mahmoud G. El Sebaei, Wafaa E. Soliman, Noushi Zaidi, Rina Tripathi, Sana Nafees, Mohammad Rumman, Durdana Yasin

**Affiliations:** 1 Department of Biosciences, Faculty of Science, Integral Centre of Excellence for Interdisciplinary Research (ICEIR), Integral University, Lucknow 226026, India; 2 Department of Bioengineering, Integral University, Lucknow 226026, India; 3 CSIR- Central Institute of Medicinal and Aromatic Plants (CSIR-CIMAP), Lucknow 226015, India; 4 Department of Biomedical Sciences, College of Clinical Pharmacy, King Faisal University, Al Ahsa 31982, Saudi Arabia; mabdelhamid@kfu.edu.sa (M.G.E.S.); weahmed@kfu.edu.sa (W.E.S.); 5 Department of Chemistry, College of Science, King Faisal University, Al-Ahsa 31982, Saudi Arabia; nzaidi@kfu.edu.sa; 6 Department of Pharmacy Practice, College of Clinical Pharmacy, King Faisal University, Al Ahsa 31982, Saudi Arabia; rinadesai@yahoo.co.in

**Keywords:** Parkinson’s disease, cyanobacteria, monoamine oxidase-B, molecular docking, molecular dynamics simulation, in silico drug discovery, neurodegeneration

## Abstract

**Background/Objectives:** Parkinson’s disease (PD) is a progressive neurodegenerative disorder characterized by dopaminergic neuronal loss and impaired motor and non-motor functions. Current therapies provide symptomatic relief but do not prevent disease progression, highlighting the need for novel neuroprotective agents. Cyanobacteria-derived bioactive compounds possess diverse pharmacological properties, including antioxidant and anti-inflammatory activities, making them potential therapeutic candidates. This study aimed to identify potential monoamine oxidase B (MAO-B) targeting cyanobacterial compounds using computational approaches. **Methods:** Forty-five cyanobacterial bioactive compounds were screened from the literature and databases based on their antioxidant and anti-inflammatory properties. Molecular docking against MAO-B was performed using PyRx, and interactions were analyzed using BIOVIA Discovery Studio. The top-ranked compounds were evaluated through ADMET profiling and 100 ns molecular dynamics simulations using the CHARMM36 force field with Swiss-Param-based ligand parameterization. **Results:** Among the screened compounds, Scytonemin (−11.9 kcal/mol), Calothrixin B (−11.7 kcal/mol), and Zeaxanthin (−11.5 kcal/mol) demonstrated the predicted binding affinity toward MAO-B. ADMET analysis revealed distinct pharmacokinetic profiles among the selected compounds. ADMET analysis showed favorable GI absorption and BBB permeability for Calothrixin B, whereas Scytonemin and Zeaxanthin showed low GI absorption and no predicted BBB permeability. Molecular dynamics simulations indicated stable ligand–protein interactions throughout the simulation period. **Conclusions:** The integrative computational analysis identified Scytonemin, Calothrixin B, and Zeaxanthin as potential MAO-B-interacting candidates. Based on the combined docking, ADMET, and molecular dynamics profiles, Calothrixin B demonstrated the most favorable overall computational profile, followed by Scytonemin and Zeaxanthin, highlighting all three compounds as candidates for further investigation in MAO-B-targeted drug discovery. Further in vitro and in vivo studies are required to validate their MAO-B inhibitory, neuroprotective, and pharmacokinetic potential.

## 1. Introduction

Parkinson’s disease (PD) is a progressive neurodegenerative condition primarily marked by the targeted loss of dopaminergic neurons in the substantia nigra pars compacta. This leads to motor symptoms such as bradykinesia, stiffness, tremors, and balance issues, along with non-motor symptoms like cognitive decline and mood changes [1]. The reduction in dopamine levels results in impaired dopaminergic signaling, which is central to the PD pathophysiology and significantly contributes to its global impact [2]. While the precise cause of PD is complex and not completely understood, oxidative stress, mitochondrial dysfunction, and abnormal protein buildup are among the widely recognized pathological mechanisms involved in the degeneration of dopaminergic neurons [3]. Pharmacological treatment of PD includes levodopa, dopamine agonists, MAO-B and COMT inhibitors, NMDA receptor antagonists, anticholinergics, and adenosine A2A receptor antagonists, each with distinct therapeutic benefits and limitations [4]. Among these targets, MAO-B is particularly relevant because it regulates dopamine metabolism, and its inhibition can increase dopamine availability while reducing dopamine-associated oxidative metabolism. Unlike dopamine receptor agonists, which primarily compensate for impaired dopaminergic neurotransmission, MAO-B inhibition directly targets dopamine catabolism and has a biologically plausible basis for reducing oxidative stress [5]. Compared with NMDA receptor antagonism, which mainly modulates glutamatergic dysfunction and dyskinesia [6], MAO-B inhibition is more directly associated with dopamine metabolism and the dopaminergic deficit in PD.

Monoamine oxidases (MAOs) are flavin-containing enzymes localized on the outer mitochondrial membrane that catalyzes the oxidative deamination of monoamine neurotransmitters including dopamine, serotonin, and noradrenaline [7]. Its two isoforms MAO-A and MAO-B are distinguished by substrate and inhibitor specificity. MAO-B, in particular, is abundantly expressed in glial cells in the brain and preferentially catalyzes the oxidation of dopamine and phenylethylamine. Hydrogen peroxide and other reactive oxygen species (ROS) are produced through the catalytic activity of MAO-B, contributing to oxidative stress and neuronal damage in PD. Moreover, it has been reported that protoxins such as 1-methyl-4-phenyl-1,2,3,6-tetrahydropyridine (MPTP) are metabolized by MAO-B into neurotoxic metabolites that induce parkinsonism in experimental models, further supporting its pathological relevance [8].

Given this central role of MAO-B in dopamine catabolism and oxidative stress, selective inhibition of MAO-B has emerged as a validated therapeutic strategy for symptomatic management of PD. Medications like Safinamide, a highly selective, reversible MAO-B inhibitor, and rasagiline and selegiline, which are irreversible MAO-B inhibitors used in clinical settings, work by decreasing dopamine degradation, thereby enhancing its availability. These drugs have been effective in alleviating motor symptoms and minimizing the “off” periods associated with levodopa. Nonetheless, the prolonged use of existing MAO-B inhibitors might be linked to limited neuroprotective benefits and potential side effects such as headache, dizziness, insomnia, orthostatic hypotension, and in some cases worsening dyskinesia or hallucinations, reflecting the limits of symptomatic MAO-B inhibition and leading to ongoing research aimed at developing new inhibitors with improved effectiveness and safety [9].

Natural products are derived from diverse biological sources including plants, microorganisms such as bacteria, fungi and cyanobacteria, and marine organisms like algae, sponges and corals, as well as animals and insects that produce bioactive compounds with medicinal value. Various types of natural compounds, such as flavonoids, coumarins, alkaloids, and other secondary metabolites, have demonstrated potential in inhibiting MAO-B, thereby broadening the chemical landscape for discovering new leads beyond synthetic frameworks [10]. Moreover, compounds derived from natural products often display structural diversity and biological significance, which are beneficial for targeting intricate enzymes like MAO-B. Some examples of a natural product derived drug is lovastatin, isolated from the fungus *Aspergillus terreus*, which is widely used in cardiovascular disease management [11]. Metformin is an antidiabetic drug that was originally inspired by compounds found in *Galega officinalis* [12]. Levodopa (L-DOPA) is a dopamine precursor used to treat Parkinson’s disease that is naturally found in *Mucuna pruriens* (velvet bean) [13]. Brentuximab vedotin, developed from the cyanobacterial peptide dolastatin-10, is an antibody–drug conjugate used in Hodgkin lymphoma and anaplastic large cell lymphoma causing microtubule disruption and cancer cell death [14].

Among these, cyanobacteria, referred to as blue-green algae, are a varied group of photosynthetic prokaryotes that generate a wide range of secondary metabolites with diverse biological activities. These metabolites include alkaloids, terpenes, peptides, and polyketides, which have been shown to possess bioactivities such as antioxidant [15] and anti-inflammatory properties [16]. Despite their potential, compounds derived from cyanobacteria have not been thoroughly assessed as MAO-B inhibitors in the context of PD. With the growing interest in natural product based MAO inhibitors, cyanobacterial metabolites represent an unexploited reservoir of chemical entities that may yield novel therapeutic leads for PD [17]. To explore the therapeutic potential of cyanobacterial compounds in the management of Parkinson’s disease, this study adopts an in silico approach. Computational techniques, including molecular docking and molecular dynamics simulation, offer critical insights into the interactions between bioactive compounds and target proteins implicated in neurodegeneration [18,19]. These methods facilitate the identification of promising lead compounds exhibiting favorable binding affinities and interaction profiles, thereby supporting their selection for further experimental validation.

## 2. Results and Discussion

### 2.1. Molecular Docking

**Figure 1:**
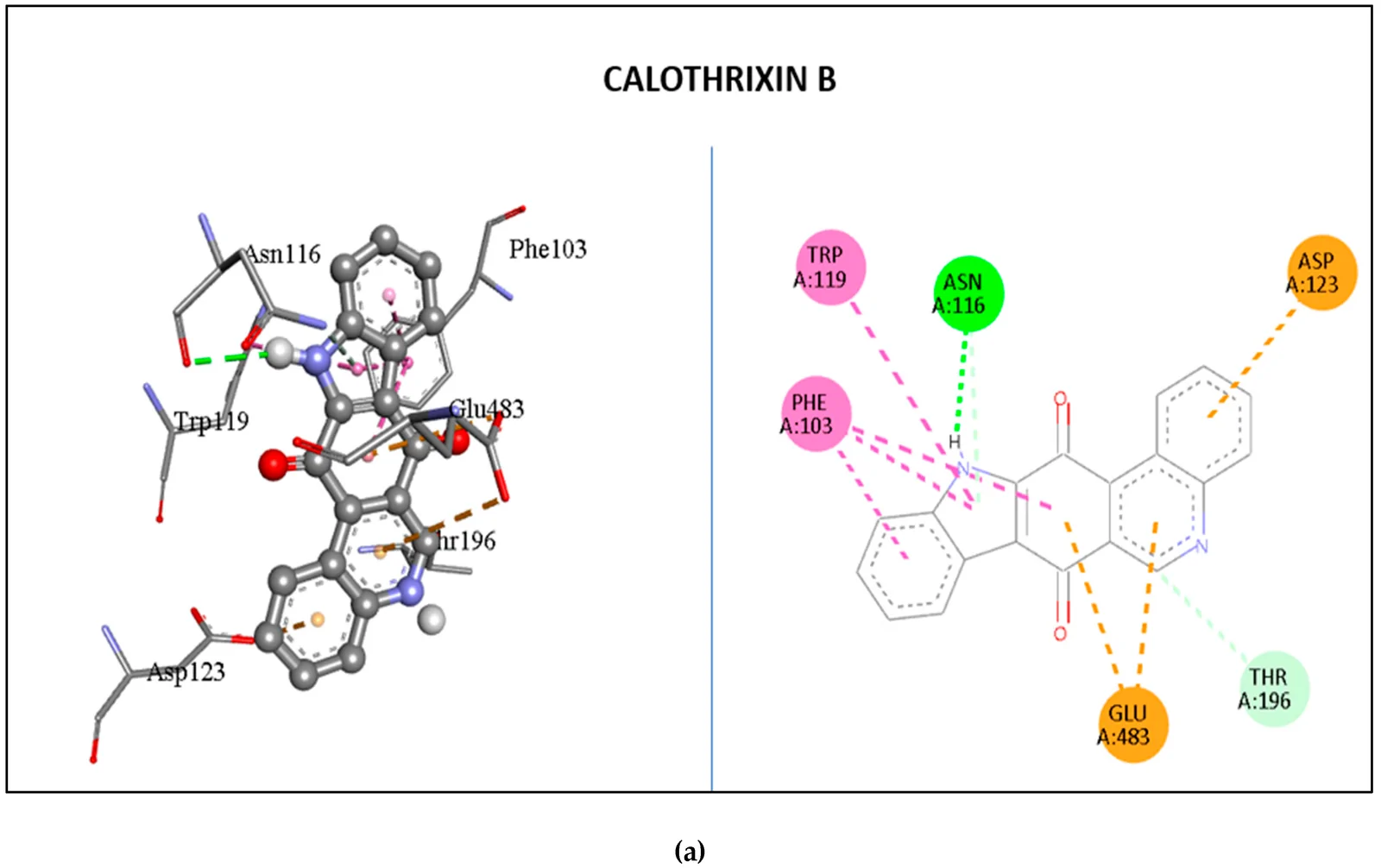

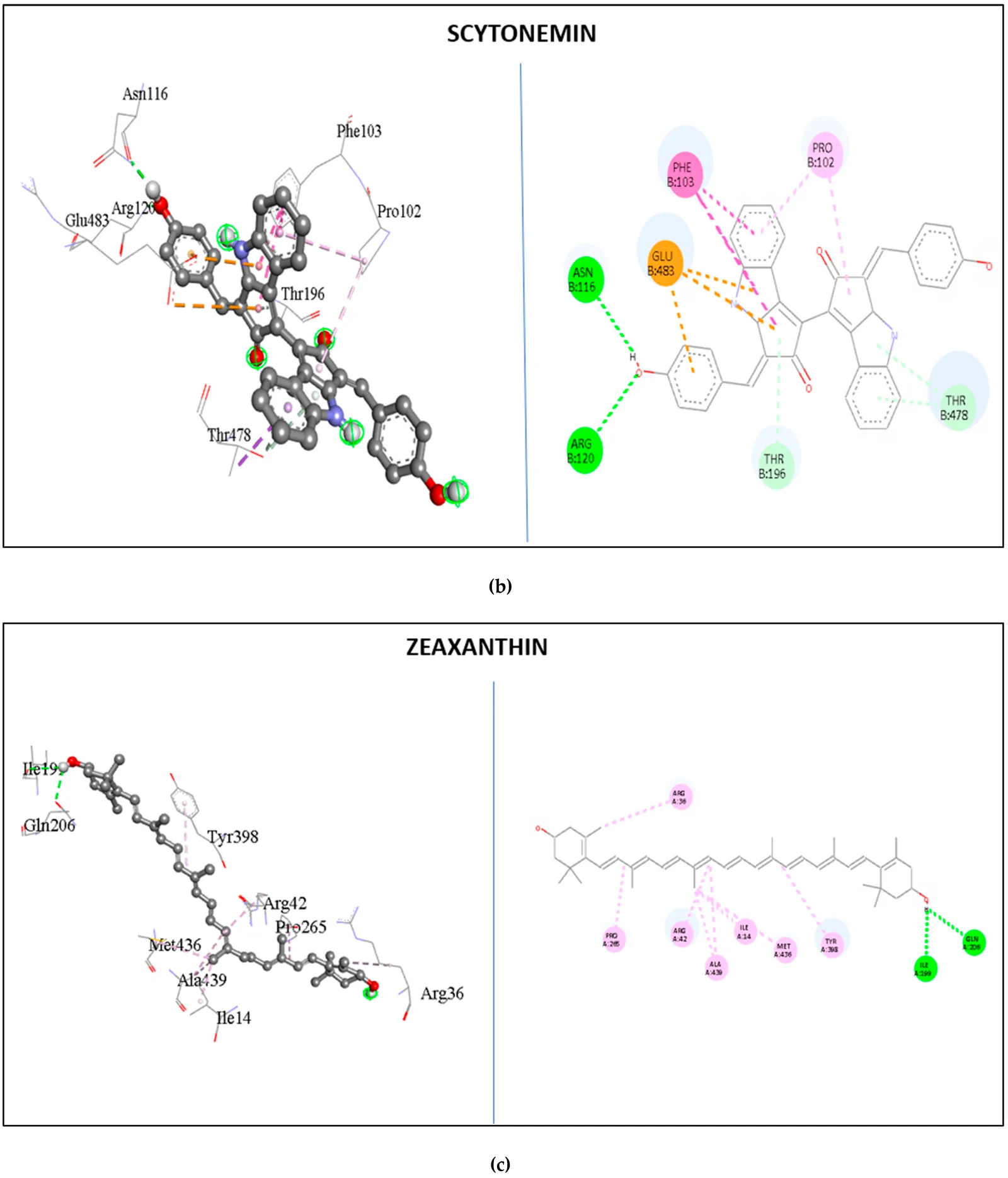

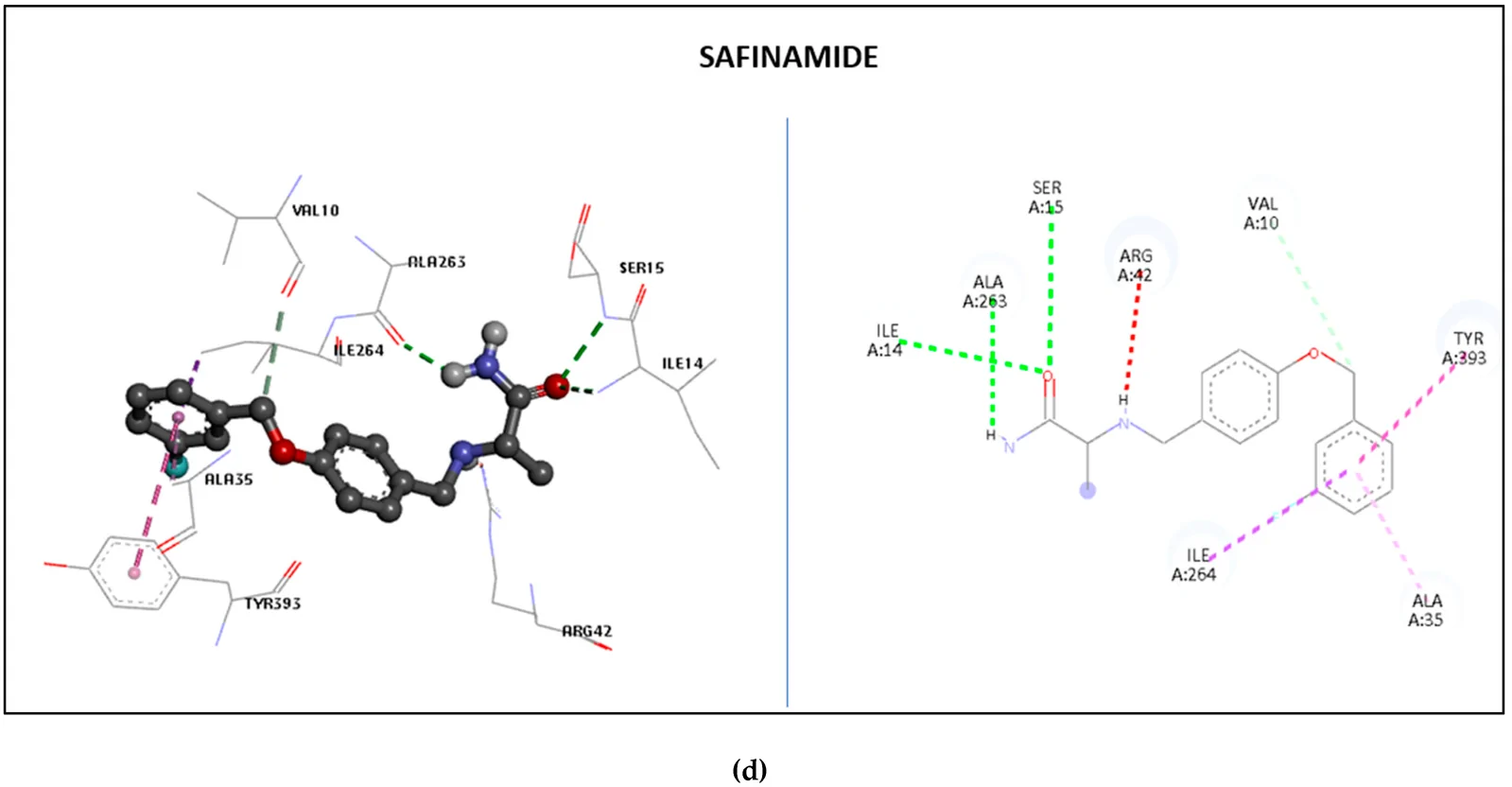
A 2D and 3D visualization of intermolecular interactions of (**a**) Calothrixin B, (**b**) Scytonemin, (**c**) Zeaxanthin and (**d**) Safinamide (control) bound with MAO-B (PDB code: 1GOS).

Forty-five ligands from PubChem were considered for the docking process. The PDB format of the drug was chosen for preparing the binding site. Molecular docking was carried out to predict the binding affinities and potential inhibitory interactions, and the docking scores are expressed as kcal/mol [20], as presented in Table 1 and Figure 1. The top three ligands with least binding energy were: −11.9 (Scytonemin) > −11.7 (Calothrixin B) > −11.5 (Zeaxanthin). Analysis of the molecular docking results revealed that all tested compounds exhibited strong binding affinity toward the target receptor MAO-B compared with the control ligand. The docking scores of the selected ligands ranged from −11.9 to −11.5 kcal/mol, whereas the control compound Safinamide showed a comparatively lower binding affinity of −9.5 kcal/mol (Supplementary File S3).

The control compound Safinamide formed three conventional hydrogen bonds with ILE1, SER15, and ALA263, with interaction distances of 3.11, 2.86, and 2.46 Å, respectively. In addition, a carbon–hydrogen bond was observed with VAL10 (3.59 Å). Several hydrophobic interactions were also identified with ILE264, TYR393, and ALA35, involving π–sigma, π–π T-shaped, and π–alkyl interactions, respectively, with distances ranging from 3.63 to 4.89 Å. These hydrogen-bonding and hydrophobic interactions may contribute to stabilization of Safinamide within the MAO-B binding pocket. Calothrixin B demonstrated a more favorable predicted docking scores of −11.7 kcal/mol. It formed two hydrogen bonds with ASN116 (conventional hydrogen bond) and THR196 (carbon–hydrogen bond). Furthermore, electrostatic π–anion interactions were observed with ASP123 and GLU483. Additional stabilization occurred through hydrophobic interactions with PHE103 and TRP119, including π–π stacked and π–π T-shaped interactions. Among the tested compounds, Scytonemin showed the highest docking score of −11.9 kcal/mol. It formed multiple hydrogen bonds with ARG120, ASN116, THR196, and THR478, including both conventional hydrogen bonds and π-donor hydrogen bonds. Electrostatic π–anion interaction was also observed with GLU483. In addition, hydrophobic interactions with PHE103 (π–π stacked) and PRO102 (π–alkyl) further stabilized the ligand–receptor complex. Zeaxanthin also exhibited strong binding with a docking score of −11.5 kcal/mol. It formed two conventional hydrogen bonds with ILE199 and GLN206, as shown in Table 2. Several hydrophobic interactions were observed with residues ARG42, PRO265, ALA439, ILE14, MET436, ARG36, and TYR398, mainly through alkyl and π–alkyl interactions, which help maintain the ligand in the active site. The relatively large molecular size and hydrophobic character of carotenoids may influence molecular docking scores through increased molecular contact surface and hydrophobic interactions. As a result, higher docking scores for Zeaxanthin should be regarded with caution and not as direct evidence of improved experimental binding affinity. In this study, docking scores were used primarily as an initial computational screening parameter and were subsequently evaluated in conjunction with ADMET profiling and molecular dynamics simulations to provide a more comprehensive assessment of the ligand–target interactions.

The results suggest that Scytonemin, Calothrixin B, and Zeaxanthin possess stronger binding affinities and more extensive molecular interactions with the receptor compared to the control compound Safinamide. Among them, Scytonemin appears to be the most promising candidate due to its highest binding affinity and multiple stabilizing interactions, indicating its potential as a candidate for further experimental evaluation of the MAO-B.

### 2.2. ADMET Results

#### 2.2.1. Physicochemical and SwissADME Bioavailability Comparison

The physicochemical descriptors that influence oral bioavailability, including molecular weight, hydrogen bond donors and acceptors, lipophilicity, topological polar surface area (TPSA), and molecular flexibility, were evaluated for the selected cyanobacterial compounds and compared with the reference compound Safinamide (Table 3). According to Lipinski’s Rule of Five, compounds with molecular weight ≤ 500 g·mol^−1^, hydrogen bond donors ≤ 5, hydrogen bond acceptors ≤ 10, and LogP ≤ 5 generally exhibit favorable membrane permeability and oral bioavailability [21]. Additionally, molecular flexibility and polarity, represented by rotatable bonds and TPSA, further influence drug absorption and transport across biological membranes.

Within this framework, Calothrixin B demonstrated a favorable drug-likeness profile, with a molecular weight of 298.29 g·mol^−1^, three hydrogen bond acceptors, and one hydrogen bond donor, all within the recommended limits of Lipinski’s rule of five. Its moderate lipophilicity (Consensus LogP 2.71) and TPSA of 62.82 Å^2^ suggest a suitable balance between hydrophobicity and polarity, which may support membrane permeability while maintaining reasonable physicochemical properties. The absence of rotatable bonds indicates a relatively rigid molecular structure, which may reduce conformational flexibility and potentially favor stable interactions with the target binding site. Furthermore, Calothrixin B exhibited a bioavailability score of 0.55, indicating a moderate predicted probability of oral bioavailability. Overall, these physicochemical characteristics suggest that Calothrixin B possesses a favorable drug-like profile.

Scytonemin exhibited a relatively higher molecular weight of 544.55 g·mol^−1^, exceeding the conventional 500 g·mol^−1^ threshold of Lipinski’s rule of five. It possessed six hydrogen bond acceptors and four hydrogen bond donors, while its three rotatable bonds indicated moderate conformational flexibility. The compound showed high lipophilicity, with a Consensus LogP of 5.80, together with a TPSA of 99.32 Å^2^, suggesting increased hydrophobic character and relatively greater polarity compared with Calothrixin B. Scytonemin showed one Lipinski violation, primarily attributable to its high molecular weight. Nevertheless, its predicted bioavailability score of 0.55 indicates a moderate probability of oral bioavailability. Although these properties suggest some limitations in conventional drug-likeness, particularly with respect to molecular weight and lipophilicity, the compound may still warrant further investigation based on its biological activity and predicted target interactions.

Zeaxanthin displayed a molecular weight of 568.87 g·mol^−1^, which exceeds the conventional molecular-weight limit of Lipinski’s rule of five. It contained two hydrogen bond acceptors and two hydrogen bond donors, while its ten rotatable bonds indicated substantial conformational flexibility. The compound exhibited markedly high lipophilicity, with a Consensus LogP of 9.83, whereas its relatively low TPSA of 40.46 Å^2^ reflects its predominantly hydrophobic character. Zeaxanthin showed two Lipinski violations, which is consistent with its high molecular weight and highly lipophilic nature. Its bioavailability score of 0.17 was considerably lower than those of Calothrixin B, Scytonemin, and Safinamide, suggesting a lower predicted probability of oral bioavailability. These physicochemical characteristics indicate that although Zeaxanthin may possess favorable hydrophobic interactions with protein binding sites, its high molecular weight, excessive lipophilicity, and low predicted bioavailability may represent important drug-development limitations.

The reference compound Safinamide exhibited a molecular weight of 302.34 g·mol^−1^, four hydrogen bond acceptors, and two hydrogen bond donors, all within the generally accepted limits of Lipinski’s rule of five. Its moderate Consensus LogP of 2.56 and TPSA of 64.35 Å^2^ indicate a balanced combination of lipophilicity and polarity that may support membrane permeability and favorable physicochemical behavior. The presence of seven rotatable bonds suggests greater conformational flexibility compared with Calothrixin B. Safinamide showed no Lipinski violations and exhibited a bioavailability score of 0.55, indicating a moderate predicted probability of oral bioavailability. Overall, its physicochemical characteristics are consistent with a favorable drug-like profile and provide an appropriate reference for comparison with the selected cyanobacterial compounds. Overall, the analysis indicates that Calothrixin B exhibits the most favorable and balanced physicochemical profile among the investigated cyanobacterial compounds, characterized by moderate molecular weight, appropriate polarity, moderate lipophilicity, absence of Lipinski violations, and a bioavailability score comparable to the reference compound Safinamide. In contrast, Scytonemin and Zeaxanthin showed comparatively greater drug-likeness limitations, particularly with respect to molecular weight, lipophilicity, and Lipinski violations, with Zeaxanthin additionally exhibiting a lower predicted bioavailability score.

The bioavailability radar predicted by SwissADME provides a graphical overview of six physicochemical parameters that influence oral drug-likeness: lipophilicity (LIPO), molecular size (SIZE), polarity (POLAR), solubility (INSOLU), saturation (INSATU), and molecular flexibility (FLEX) [22]. The pink region in the radar indicates the ideal range of six key parameters: lipophilicity (LIPO: −0.7 < XLOGP3 < 5.0), molecular size (SIZE: 150 g/mol < molecular weight < 500 g/mol), polarity (POLAR: 20 Å^2^ < TPSA < 130 Å^2^), solubility (INSOLU: −6 < Log S (ESOL) < 0), saturation (INSATU: 0.25 < fraction Csp^3^ < 1), and flexibility (FLEX: number of rotatable bonds < 9) [22]. Compounds whose radar plots fall largely within this pink shaded region are predicted to possess favorable physicochemical properties for oral bioavailability.

**Figure 2:**
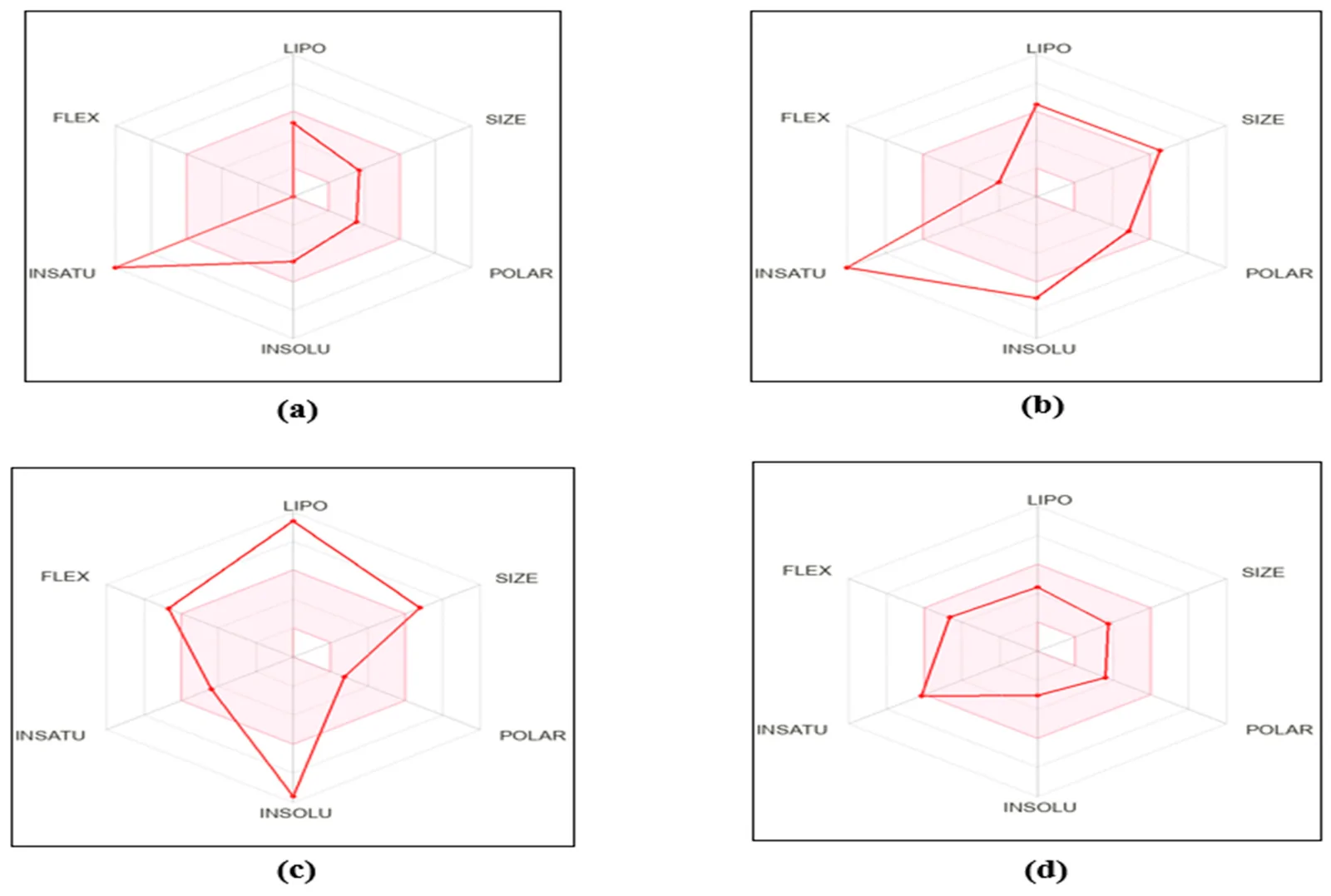
Bioavailability radar for (**a**) Calothrixin B, (**b**) Scytonemin, (**c**) Zeaxanthin, and (**d**) Safinamide.

The radar profile shown in Figure 2a indicates that Calothrixin B falls within the optimal range for lipophilicity, molecular size, polarity, solubility, and molecular flexibility, suggesting suitable physicochemical characteristics for oral absorption. The compound exhibits moderate lipophilicity and acceptable polarity, which may support membrane permeability and pharmacokinetic stability. Additionally, the low number of rotatable bonds reflects limited molecular flexibility, a feature often associated with improved binding stability. However, the INSATU parameter lies outside the optimal region, indicating a relatively low fraction of sp^3^-hybridized carbons and a higher degree of molecular unsaturation, likely due to the presence of aromatic rings in the structure. Despite this deviation, the overall radar profile suggests that Calothrixin B possesses physicochemical properties largely consistent with drug-like molecules and may exhibit favorable oral bioavailability characteristics.

Figure 2b indicates that radar profile of Scytonemin has acceptable positioning of the lipophilicity and polarity parameters, although its lipophilicity approaches the upper boundary of the favorable region, which is consistent with its relatively high Consensus LogP of 5.80. The molecular size extends toward or beyond the optimal region, corresponding to its relatively high molecular weight of 544.55 g·mol^−1^. The flexibility parameter remains within the favorable region, indicating a comparatively restricted degree of conformational flexibility despite the presence of three rotatable bonds. In contrast, the INSOLU parameter indicates reduced aqueous solubility, which may adversely influence dissolution and absorption. The INSATU parameter extends markedly outside the optimal region, reflecting the highly unsaturated and aromatic character of Scytonemin. Overall, the radar profile suggests that Scytonemin possesses several physicochemical characteristics compatible with drug-likeness; however, its relatively large molecular size, high lipophilicity, limited solubility, and pronounced unsaturation may impose limitations on its oral bioavailability and pharmacokinetic properties (Supplementary File S2).

As shown in Figure 2c’s radar plot, Zeaxanthin exhibits the least favorable SwissADME radar profile among the investigated cyanobacterial compounds. The lipophilicity parameter extends substantially beyond the optimal region, which is consistent with its very high Consensus LogP of 9.83 and pronounced hydrophobic character. Its molecular size is positioned toward the upper limit of the favorable region, reflecting its high molecular weight of 568.87 g·mol^−1^. Although the polarity parameter remains relatively low, the INSOLU parameter extends markedly outside the optimal region, indicating poor predicted aqueous solubility associated with its highly lipophilic nature. The flexibility parameter also approaches the outer boundary of the radar, which is consistent with the presence of ten rotatable bonds and substantial conformational flexibility. The INSATU parameter remains outside or close to the lower boundary of the favorable region, reflecting the highly unsaturated structure characteristic of carotenoids. Collectively, the radar profile indicates that Zeaxanthin possesses substantial deviations from the optimal physicochemical space, particularly with respect to lipophilicity, solubility, molecular size, and flexibility, which may contribute to its comparatively low predicted oral bioavailability.

In Figure 2d, Safinamide, used as the reference compound, displays a comparatively balanced radar profile, with most of its physicochemical parameters positioned within the favorable region. Its lipophilicity and molecular size fall within the optimal range, which is consistent with its moderate Consensus LogP of 2.56 and molecular weight of 302.34 g·mol^−1^. The polarity parameter also remains within the favorable region, indicating an appropriate balance between polarity and lipophilicity. The INSOLU parameter is positioned relatively close to the optimal region, suggesting comparatively favorable aqueous solubility characteristics. Its flexibility parameter remains within the acceptable range, although it is higher than that of Calothrixin B, which is consistent with the presence of seven rotatable bonds. The INSATU parameter is also positioned closer to the favorable region than those of the highly unsaturated cyanobacterial compounds. Overall, the radar profile of Safinamide demonstrates a well-balanced physicochemical distribution, which is consistent with its established drug-like characteristics and its suitability as the reference compound in the present analysis. These findings further validate the reliability of the SwissADME predictions and provide a useful benchmark for comparing the bioavailability profiles of the studied cyanobacterial compounds.

#### 2.2.2. In Silico Pharmacokinetics ADMET Prediction

The pharmacokinetic properties of the selected cyanobacterial compounds were predicted using SwissADME to evaluate their absorption, distribution, metabolism, and permeability characteristics [23], and potential cytochrome P450 (CYP)-mediated drug–drug interaction profiles. As summarized in Table 4, the three compounds exhibited distinct pharmacokinetic characteristics.

Calothrixin B demonstrated high gastrointestinal (GI) absorption, indicating a favorable predicted capacity for absorption following oral administration. It was predicted to be permeable across the blood–brain barrier (BBB), suggesting that Calothrixin B may have comparatively greater potential to reach the central nervous system. Furthermore, it was predicted not to be a P-glycoprotein (P-gp) substrate, which may favor intracellular retention and reduce active efflux-mediated limitations on its distribution. With respect to CYP-mediated interactions, Calothrixin B was predicted to act as an inhibitor of CYP1A2 and CYP2C9, whereas it showed no inhibitory activity toward CYP2C19 and CYP2D6. Its predicted skin permeability coefficient (log Kp) was −2.02 cm/s, indicating comparatively greater skin permeation among the three investigated compounds.

Scytonemin exhibited low GI absorption, suggesting comparatively limited absorption through the gastrointestinal tract. In contrast to Calothrixin B, Scytonemin was predicted to be non-permeable across the BBB, which may restrict its passive distribution into the central nervous system. It was also predicted not to be a P-gp substrate, indicating that P-gp-mediated efflux may not be a major determinant of its pharmacokinetic disposition. Regarding CYP-mediated interactions, Scytonemin was predicted to be inactive against CYP1A2, CYP2C19, and CYP2D6, while showing inhibitory activity toward CYP2C9. The predicted log Kp value of −5.45 cm/s indicates substantially lower skin permeability than that predicted for Calothrixin B.

Zeaxanthin showed low GI absorption and was predicted to be non-permeable across the BBB, suggesting limited gastrointestinal absorption and restricted penetration into the central nervous system under the predicted conditions. Unlike Calothrixin B and Scytonemin, Zeaxanthin was predicted to be a P-gp substrate, indicating that active P-gp-mediated efflux may contribute to its disposition and potentially limit its intracellular accumulation. Zeaxanthin was predicted to be inactive against CYP1A2, CYP2C19, and CYP2D6, but showed inhibitory activity toward CYP2C9, similar to the other two compounds. Its predicted skin permeability coefficient was −5.56 cm/s, representing the lowest skin permeability among the investigated compounds. Overall, Zeaxanthin exhibited a comparatively less favorable predicted pharmacokinetic profile, particularly with respect to GI absorption, BBB permeability, P-gp substrate status, and skin permeation.

#### 2.2.3. Predictive Acute-Toxicity Profile (Protox 3) of the Three Selected Cyanobacterial Compounds with the Control Safinamide

**Figure 3:**
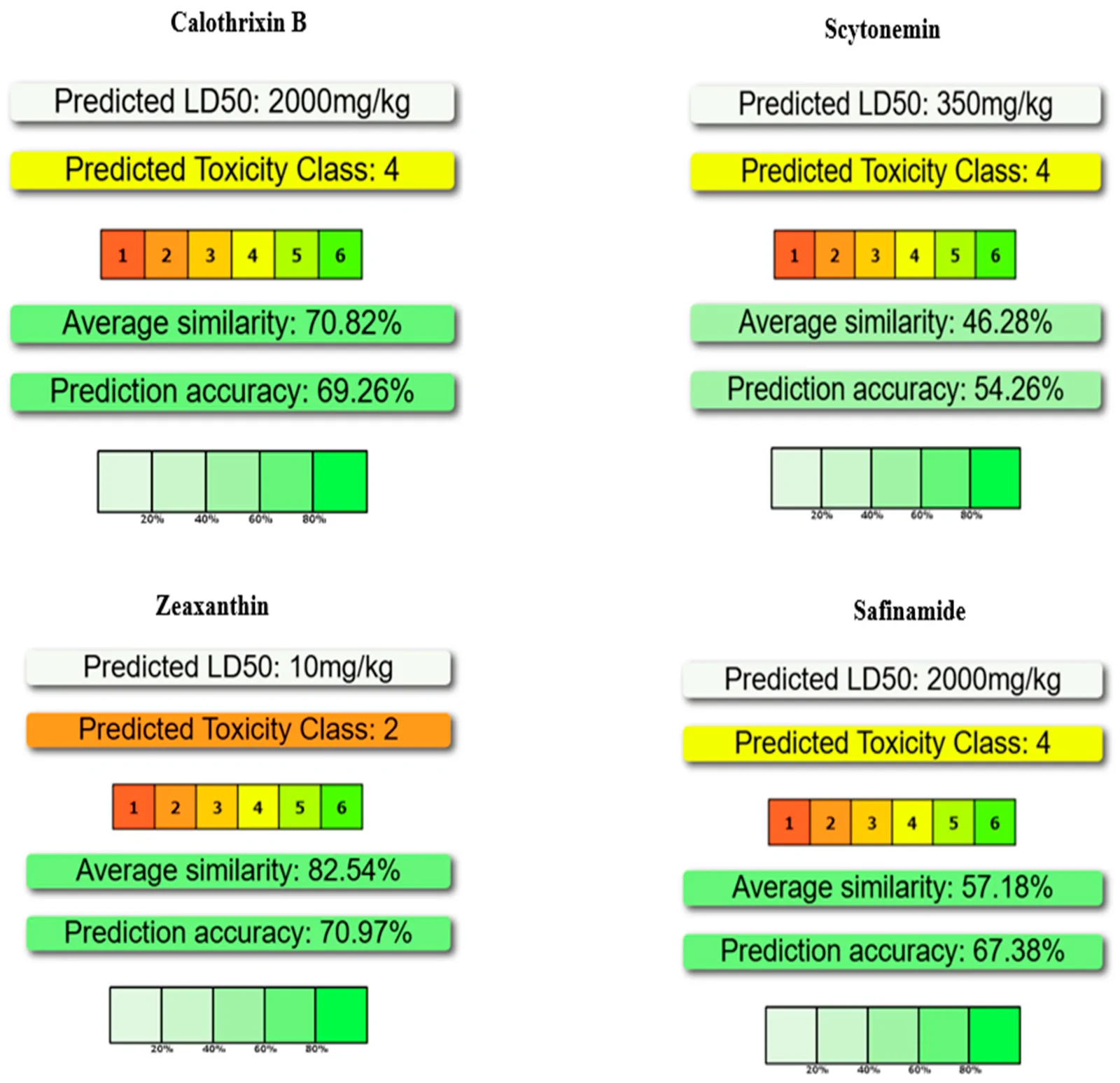
Protox 3 results: LD50, toxicity class, and prediction reliability for each compound (Calothrixin B, Scytonemin, Zeaxanthin) and the control (Safinamide).

The Protox 3 outputs in Figure 3 indicate a toxicity gradient that parallels the electrophilicity, lipophilicity, and metabolic-activation potential recorded in each scaffold. Calothrixin B (predicted LD50 ≈ 2000 mg kg^−1^, toxicity class 4) exhibited a moderate acute toxicity profile according to the in silico toxicity prediction (Supplementary File S1). As a quinone-containing indolo [3,2-j] phenanthridine alkaloid, Calothrixin B possesses an extended planar aromatic system that may facilitate biological interactions and redox activity, potentially contributing to its predicted toxicity. The predicted LD50 of approximately 2000 mg kg^−1^ places Calothrixin B in toxicity class 4, indicating moderate acute toxicity rather than a highly toxic profile. The prediction showed an average structural similarity of 70.82% and a prediction accuracy of 69.26%, suggesting a moderate-to-good confidence level in the computational estimation.

Scytonemin exhibited a lower predicted LD_50_ of approximately 350 mg/kg, while remaining within toxicity class 4. The highly conjugated and lipophilic architecture of Scytonemin may facilitate biological membrane interactions and influence its systemic disposition. However, its Protox 3 prediction was associated with a relatively lower average similarity 46.28% and prediction accuracy 54.26%, indicating that the predicted toxicity should be interpreted with greater caution.

Zeaxanthin displayed the most pronounced predicted acute toxicity among the investigated compounds, with a predicted LD_50_ of approximately 10 mg/kg and assignment to toxicity class 2. This prediction represents a substantial difference from the other compounds and suggests a potentially higher acute toxicity liability according to the Protox 3 model. Its extended polyene chain and highly lipophilic character can influence membrane partitioning, tissue distribution, and metabolic susceptibility, potentially contributing to the predicted toxicity profile. Nevertheless, Zeaxanthin showed a relatively high average similarity (82.54%) and prediction accuracy (70.97%), providing comparatively stronger computational support for the prediction.

Safinamide (predicted LD50 ≈ 2000 mg kg^−1^, toxicity class 4) exhibited a moderate acute toxicity profile in the in silico prediction. The compound contains a fluorinated aromatic moiety together with ether, amine, and carboxamide functionalities, providing a combination of lipophilic and polar structural features that may influence its systemic disposition and toxicity. The predicted LD50 of approximately 2000 mg kg^−1^ places Safinamide within toxicity class 4, corresponding to comparatively lower acute toxicity than compounds with substantially smaller predicted LD50 values, although it does not indicate complete absence of toxic effects. The prediction was associated with an average structural similarity of 57.18% and a prediction accuracy of 67.38%, indicating a moderate level of confidence in the computational estimate. Although Protox 3 predicted a low LD_50_ value for Zeaxanthin and moderate prediction reliability for Scytonemin, these results should be interpreted with caution. The predictive performance of computational toxicity models may be limited for structurally complex or structurally diverse compounds that may fall outside the model’s applicability domain. Therefore, the predicted toxicity parameters were considered preliminary estimates requiring further experimental validation.

### 2.3. Molecular Dynamics Simulations

**Figure 4:**
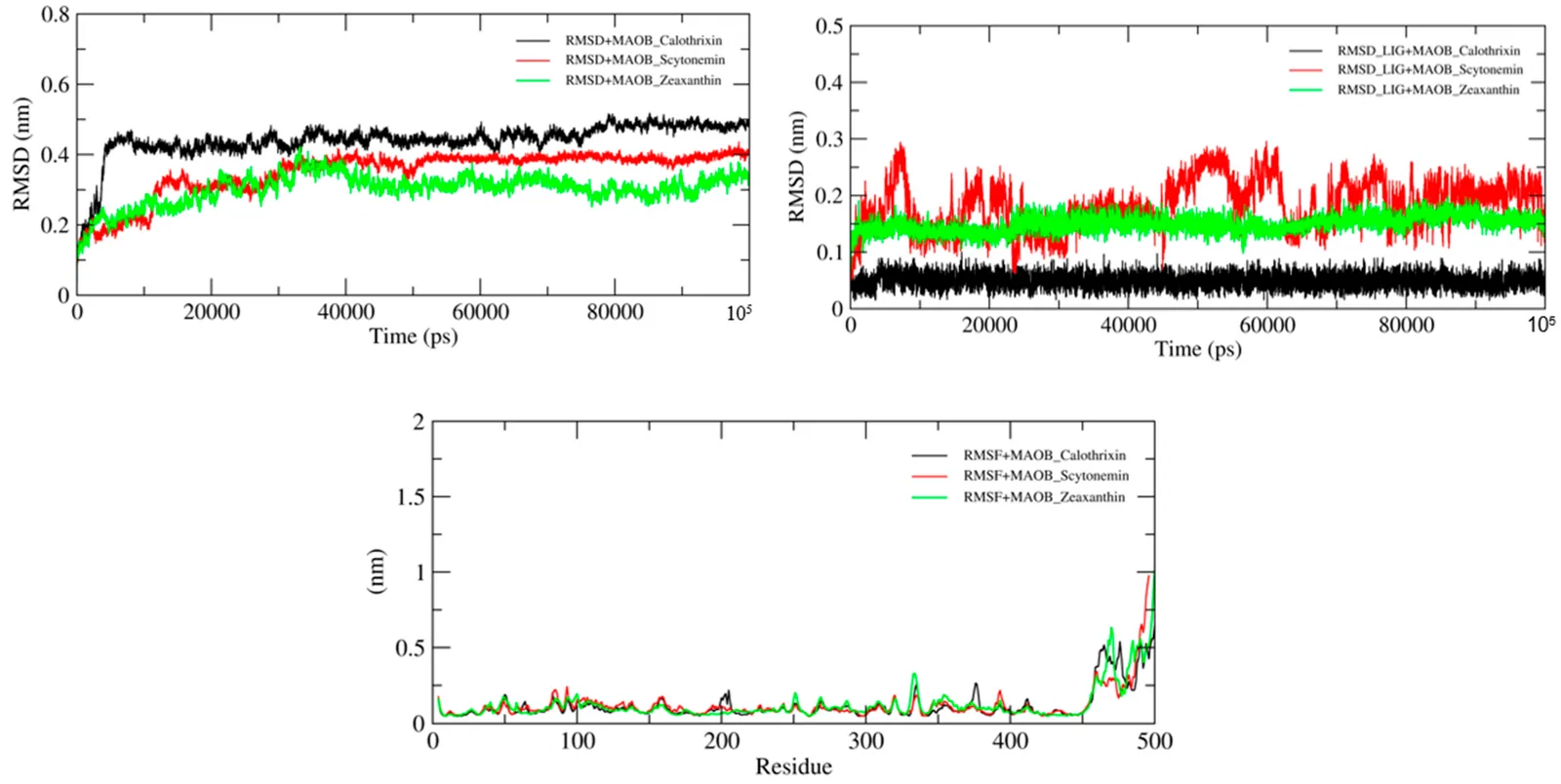
RMSD and RMSF analyses of MAO-B complexes with Calothrixin B, Scytonemin, and Zeaxanthin during a 100 ns MD simulation.

From the performed trajectory analysis, the RMSD (Root mean square deviation) backbone analysis of all three complexes illustrated that the Calothrixin complex showed a higher RMSD of >0.4 nm in comparison with the other two complexes; overall, the trajectory remains stable, with some minor fluctuations, as show in Figure 4.

The RMSD ligand analysis showed that the ligands Calothrixin and Zeaxanthin exhibit a stable trajectory whereas the ligand Scytonemin exhibits the least stable with a high fluctuating trajectory, suggesting that the ligand tends to be highly dynamic inside the pocket.

The RMSF (Root mean square fluctuation) analysis shows higher fluctuations near the residues between 460 and 500 residues after ligand binding and from the three complexes; binding of Scytonemin shows lower fluctuations, followed by Calothrixin, while binding of Zeaxanthin comparatively shows higher fluctuations nearby that region. This shows that the binding of Scytonemin and Calothrixin traps the helical region between residues 490 and 500.

**Figure 5:**
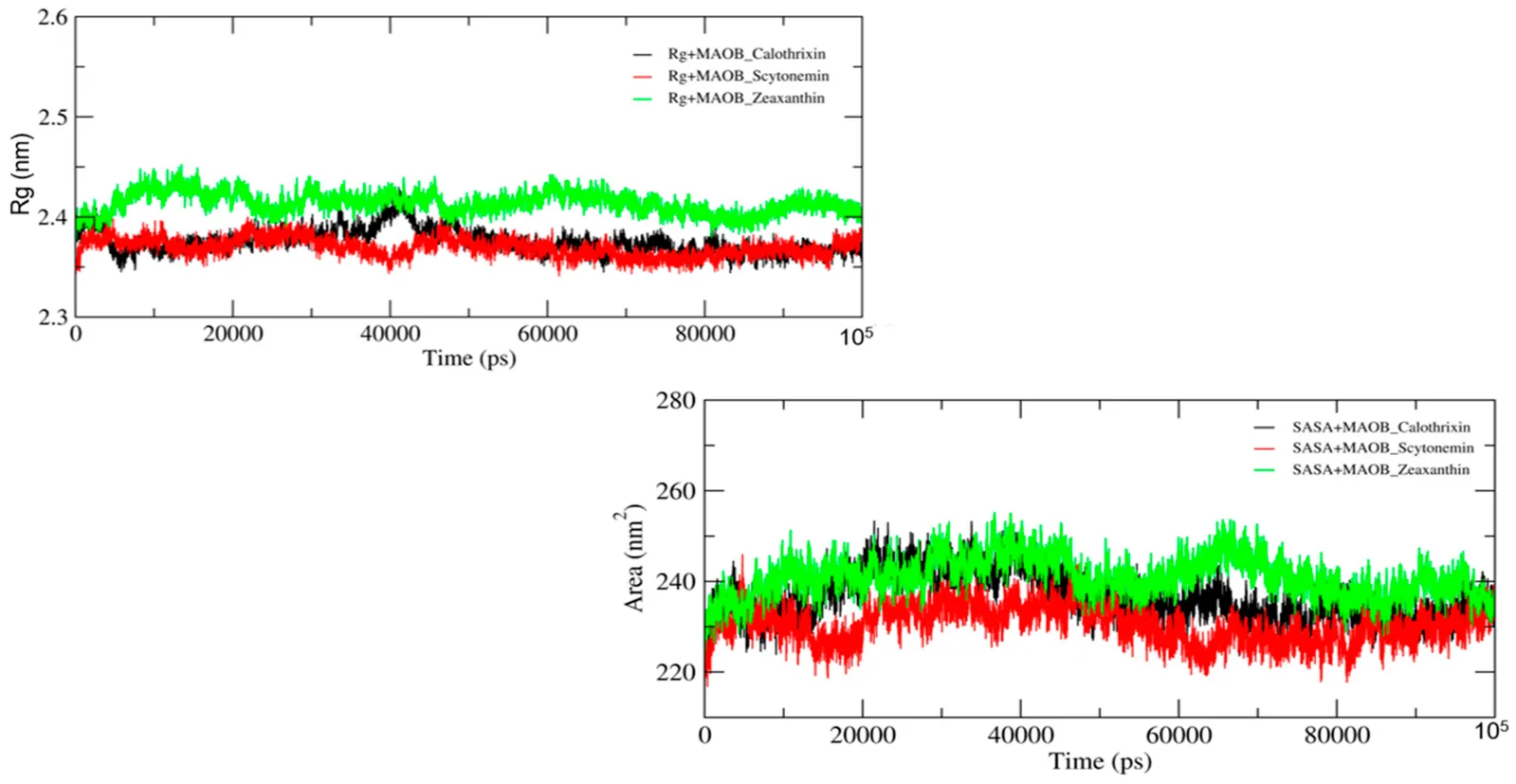
Radius of gyration (Rg) and solvent-accessible surface area (SASA) analysis of MAO-B–ligand complexes during molecular dynamics simulation.

The Radius of Gyration and SASA remain stable throughout the trajectory for all three complexes. The hydrogen bond analysis showed that all three complexes exhibit hydrogen bond interaction, with Zeaxanthin forming the greater number of hydrogen bonds (2–4 bonds); initially they were formed as lesser bonds but later around 60–100 ns they forms four stable H-bonds with the protein, followed by Scytonemin forming 2–3 bonds and then Calothrixin forming 1–2 bonds, as shown in Figure 5.

**Figure 6:**
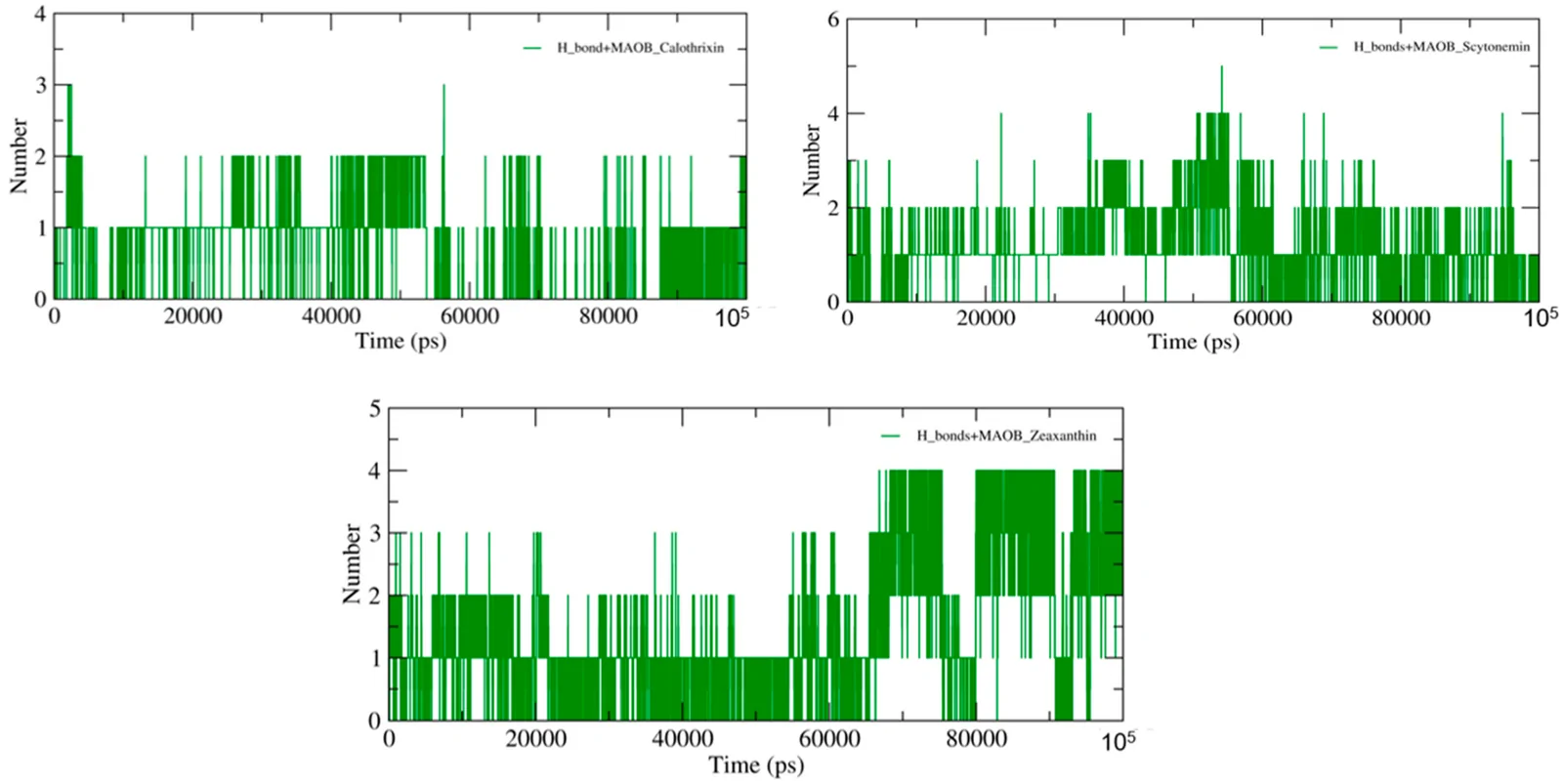
Hydrogen bond occupancy between MAO-B and Calothrixin B, Scytonemin, and Zeaxanthin during 100 ns molecular dynamics simulation.

Further, the last conformation from the MD run of the protein–ligand complex was extracted and their position and interactions were analyzed. Time-dependent hydrogen bond profiles of MAO-B complexed with Calothrixin B, Scytonemin, and Zeaxanthin over a 100 ns simulation period are shown. The plots depict the number of intermolecular hydrogen bonds formed between the protein and each ligand as a function of time. Zeaxanthin exhibits a higher and more persistent hydrogen bond occupancy (up to four hydrogen bonds), particularly during the later stages of the simulation, whereas Scytonemin forms predominantly 2–3 hydrogen bonds and Calothrixin B shows comparatively fewer interactions (1–2 hydrogen bonds), indicating differential stability and binding behavior among the complexes as shown in Figure 6. A single 100 ns MD trajectory was generated for each protein–ligand complex; therefore, the results should be interpreted cautiously. Future studies employing multiple independent simulations would provide improved statistical confidence and reproducibility.

## 3. Materials and Methods

### 3.1. Computational Tools and Software Platforms

The study was conducted using bioinformatics tools and software, including PyRx 0.8 for molecular docking [24], Biovia Discovery Studio 2025 for visualization, MolSoft https://www.molsoft.com/ (accessed on 27 October 2025). for drug-likeness evaluation, and SwissADME for in silico ADMET prediction [25]. The 64-bit Windows 10 and 32-bit Windows 7 operating systems support biological databases. Key biological databases used for ligand and protein retrieval included PubChem (http://pubchem.ncbi.nlm.nih.gov) [26], and Research Collaboratory for Structural Bioinformatics and Protein Data Bank (RCSB PDB) (http://www.rcsb.org/pdb/) [27], respectively.

### 3.2. Preparation of Ligands

The major bioactive compounds of cyanobacteria namely, Ambigol A, Ambigol B, Ambiguine H isonitrile, Ambiguine I isonitrile,, Aplysiatoxin, Astaxanthin, Barbamide, Calothrixin B, Canthaxanthin, Carazostatin, Coibacin A, Curacin A, Curacin D, Debromoaplysiatoxin, Echinenone, Ethyl tumonoate A, Fischerellin A, Fischerindole L, Gallic acid, Grenadamide, Hapalindole A, Herbamide B, Homoanatoxin A, Honaucin A, Kalkitoxin, Luteolin 7-O-glucoside (cynaroside), Lyngbic acid, Lyngbyabellin A, Lyngbyatoxin A, Merocyclophane A, Mycosporine-2-glycine, Nocuolin A, Nostocarboline, Nostocine A, Palmyramide A, Rutin, Scytonemin, Scytoscalarol, Tanikolide, Tjipanazole J, Ulongamide A, Unnarmicin D, Venturamide B, Welwitindolinone A isonitrile, and Zeaxanthin, were selected as ligands. A final library of 45 cyanobacterial bioactive compounds was selected for computational analysis, after excluding five compounds that did not meet the predefined selection criteria, including non-cyanobacterial compounds and reported neurotoxins. The list of the selected cyanobacterial compounds along with their PubChem IDs and structures is shown in Table 5. These compounds were chosen from the literature based on their reported medicinal properties such as antioxidant, anti-inflammatory, and antiapoptotic properties. Antioxidant and anti-inflammatory activities were considered during the preliminary prioritization of cyanobacterial metabolites because oxidative stress and neuroinflammation are closely interconnected pathological processes in PD. Excessive reactive oxygen species (ROS), arising from mitochondrial dysfunction, dopamine metabolism, and impaired endogenous antioxidant defenses, can promote oxidative damage to proteins, lipids, and nucleic acids and contribute to the degeneration of dopaminergic neurons [28]. In parallel, activation of microglia and the subsequent release of pro-inflammatory mediators, including IL-1β, IL-6, TNF-α, and COX-2-associated mediators, can further enhance ROS generation and neuronal injury [29]. Thus, compounds possessing antioxidant or anti-inflammatory activities were considered biologically relevant for constructing the preliminary candidate library. The 3D structures of each ligand, along with the standard drug (Safinamide) were retrieved from the PubChem database.

### 3.3. Molecular Docking

**Figure 7:**
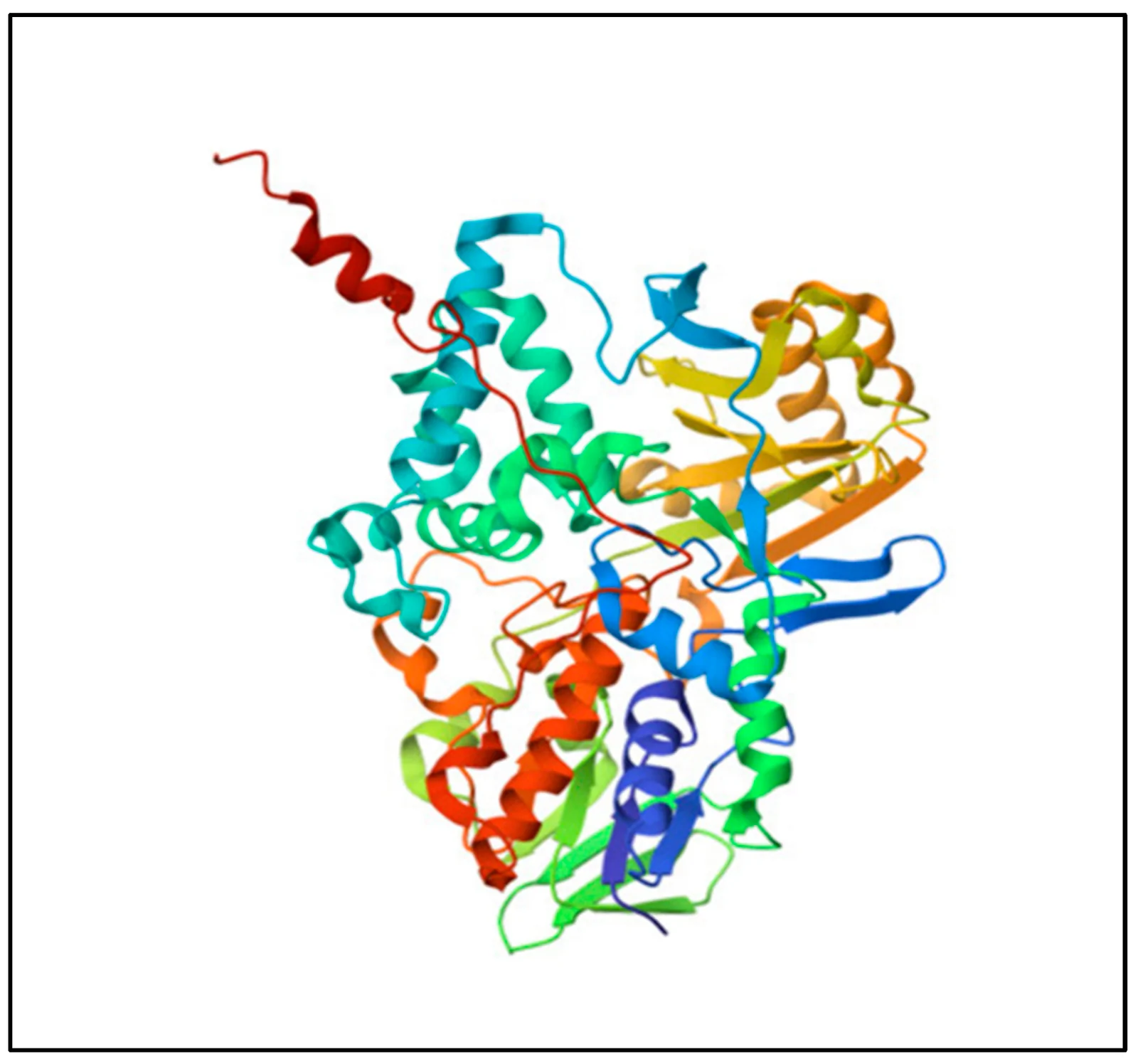
A 3-D structure of monoamine oxidase-B (PDB ID: 1GOS).

Molecular docking is a reliable method for predicting interactions between ligands (inhibitors) and receptors (enzymes), reducing the time required for in silico drug discovery [30]. The crystal structures of the target protein MAO-B (PDB ID: 1GOS) was obtained from the RCSB PDB (Figure 7). The protein structure includes water molecules, and a native ligand that were removed, while retaining the catalytically essential FAD cofactor to preserve the native architecture of the MAO-B binding site for subsequent molecular docking, using Discovery Studio 2025 to ensure accurate molecular docking [31]. The cleaned structure was saved in PDB format. Only Chain A of MAO-B was selected for docking, as it sufficiently represents the functional unit of the enzyme.

Ligand structures were obtained from PubChem [26] and energy-minimized using Open Babel implemented in PyRx (version 0.8) [24]. Both protein and ligand structures were subsequently converted into PDBQT format prior to docking. Molecular docking simulations were carried out using AutoDock Vina 1.1 integrated within PyRx to predict the binding affinity and orientation of ligands within the active site of MAO-B [32]. Blind molecular docking was performed to explore potential binding regions and interaction modes of the selected cyanobacteria-derived compounds with human MAO-B. Since the binding sites of these compounds on MAO-B have not been experimentally established, no predefined binding pocket was imposed during the initial docking analysis. A relatively large docking grid with dimensions 107.58 × 104.90 × 25.00 Å and grid center set at coordinates X = 37.4, Y = 141.2, Z = 20.7 was used to allow the ligands to explore a broad region of the receptor surface. The resulting docking poses were analyzed to identify potential ligand-binding regions and protein–ligand interactions. The docking results were considered exploratory predictions of potential binding modes rather than definitive evidence of to the canonical MAO-B catalytic or substrate-binding cavity. The exhaustiveness parameter was set to 8 to achieve a balance between computational efficiency and conformational sampling.

**Figure 8:**
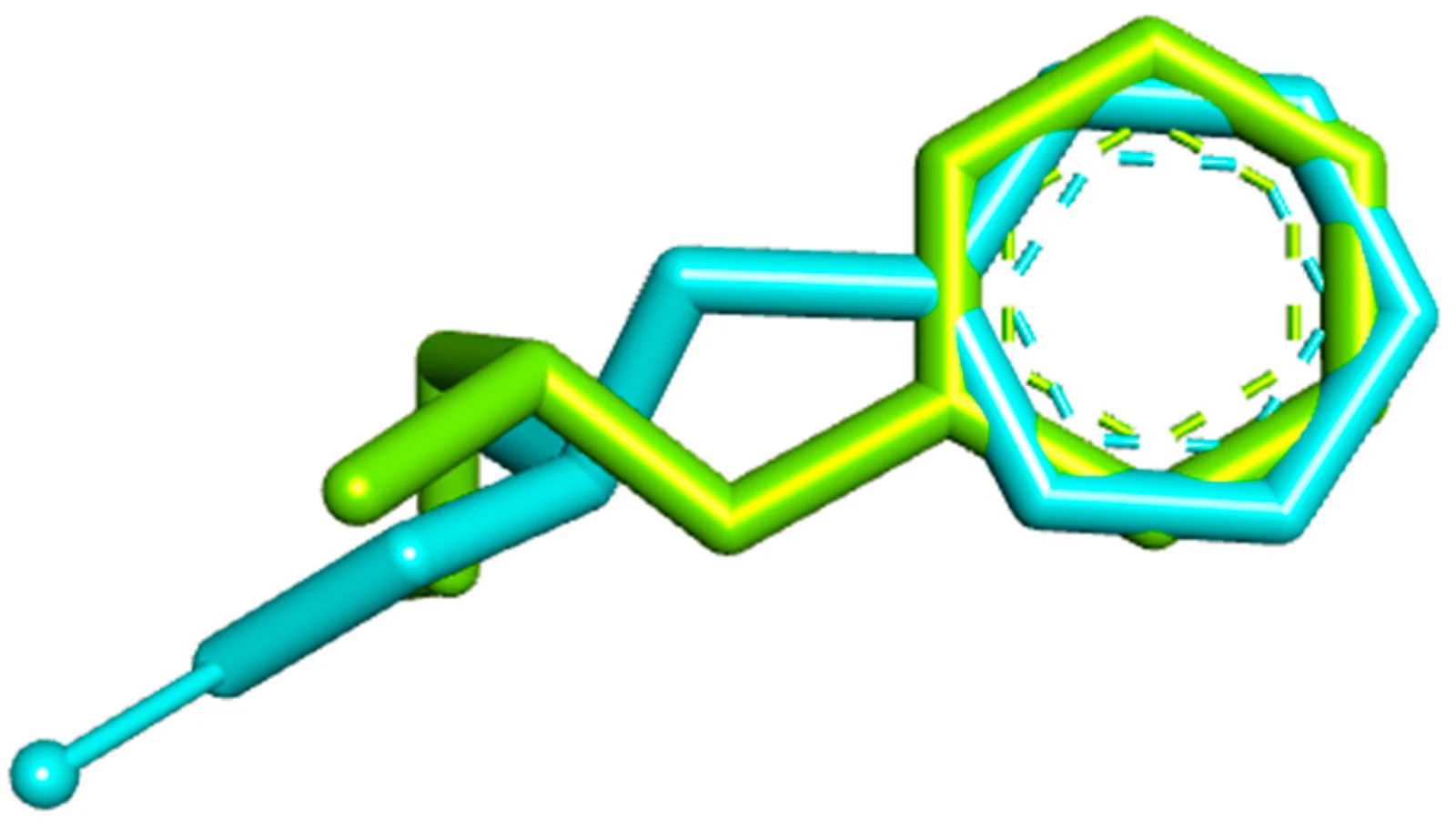
Re-docking pose with RMSD equal to 1.8147 Å (original NYP in cyan color and docked NYP in green color).

Protocol validation: The crystal structure of MAO-B (PDB ID: 1GOS), containing the catalytic cofactor flavin-adenine dinucleotide (FAD), was used for molecular docking, with FAD retained to preserve the native catalytic environment. The crystallographic inhibitor N-(E)-methyl-N-[(E)-2-propenylidene]methanaminium (NYP) was removed and subsequently redocked using the same whole-protein grid dimensions and docking parameters employed for the virtual screening of the selected cyanobacterial compounds. Thus, the validation and production docking runs were performed under identical blind-docking conditions. The efficiency of the docking procedure was assessed by superimposing the redocked NYP pose onto the crystallographic ligand pose, as shown in Figure 8. The redocked ligand exhibited a heavy-atom RMSD of 1.8147 Å, which is below the generally accepted threshold of 2.0 Å, indicating successful reproduction of the experimental binding orientation. Thus, the docking protocol was considered sufficiently reliable for subsequent virtual screening and molecular docking of the selected cyanobacterial compounds. Although successful redocking of the crystallographic ligand supported the ability of the applied docking protocol to reproduce the reported binding orientation, this validation was based on the crystallographic ligand present in 1GOS. Therefore, the present validation does not independently establish the ability of the protocol to reproduce the binding mode of a known noncovalent MAO-B inhibitor.

### 3.4. ADMET Studies

To evaluate the pharmacokinetic suitability of the selected cyanobacterial compounds, in silico ADMET (absorption, distribution, metabolism, excretion and toxicity) analysis was performed [23]. The physicochemical properties and drug-likeness parameters of the selected compounds were analyzed using the SwissADME online server [33]. Drug-likeness was assessed according to established criteria, including Lipinski’s rule of five, to determine the oral bioavailability and suitability of the compounds as potential drug candidates [34]. Assessment of these parameters is essential prior to clinical investigation to predict the drug-likeness and safety profile of candidate molecules.

### 3.5. Molecular Dynamics Simulations

Based on the molecular docking results, selected protein–ligand complexes exhibiting favorable binding orientations and docking scores were further subjected to molecular dynamics (MD) simulations to evaluate the stability, and dynamic interactions of the complexes under physiological conditions. The selected systems included PDB ID: 1GOS complexed with Scytonemin, Calothrixin B and Zeaxanthin. Each system was simulated independently for a total simulation time of 100 ns.

Molecular dynamics simulations were performed using GROMACS version 2025.2 with the CHARMM36 force field. The native FAD cofactor was retained in the MAO-B structure, and its topology and parameters were generated using the SwissParam server. During system preparation, FAD was treated as a separate parameterized cofactor, and the native covalent FAD–protein linkage was not retained in the MD simulations. The ligand parameters were generated using SwissParam2023. The protein–ligand complex was placed in a cubic simulation box with approximately 10 Å padding from the solute boundaries and solvated using the TIP3P water model. The system was neutralized by adding appropriate counterions, followed by the addition of NaCl to achieve an ionic strength of 0.15 M.

Prior to the production simulation, the solvated system underwent energy minimization using the steepest-descent algorithm, which was continued until the maximum force was reduced below 1000 kJ mol^−1^ nm^−1^. The minimized system was subsequently equilibrated in two sequential phases. Initially, a 500 ps NVT equilibration was performed at 300 K using the Nosé–Hoover thermostat to maintain the desired temperature. This was followed by a 500 ps NPT equilibration at 300 K and 1 bar, with pressure maintained using the Parrinello–Rahman barostat.

Following equilibration, a 100 ns production MD simulation was performed under NPT conditions at 300 K and 1 bar, using a 2 fs integration time step. All covalent bonds involving hydrogen atoms were constrained using the LINCS algorithm, while long-range electrostatic interactions were treated using the Particle Mesh Ewald (PME) method. The resulting trajectories were recorded at 10 ps intervals for subsequent structural and dynamical analyses, including assessment of RMSD, RMSF, radius of gyration, hydrogen bonding, and protein–ligand interaction stability.

### 3.6. Trajectory Analysis

The MD trajectories were analyzed using Grace to evaluate the dynamic stability of the protein–ligand complexes. Structural parameters including root mean square deviation (RMSD), root mean square fluctuation (RMSF), radius of gyration (Rg), and solvent-accessible surface area (SASA) were calculated. Protein–ligand interaction stability was monitored by evaluating hydrogen bonds and distance-based contacts throughout the simulation time.

## 4. Conclusions

The present study employed an integrative in silico approach to identify potential MAO-B-interacting compounds from cyanobacteria relevant to Parkinson’s disease. Molecular docking of Forty-five cyanobacterial compounds identified Scytonemin, Calothrixin B, and Zeaxanthin among the compounds with the most favorable predicted docking scores against MAO-B. These results suggested favorable predicted interactions within the MAO-B active-site region. Subsequent MD simulations provided further insights into the structural stability and dynamic behavior of the MAO-B–ligand complexes. Backbone RMSD analysis indicated that all three complexes maintained overall structural stability throughout the simulation, although differences in ligand and residue fluctuations were observed. Analysis of the final MD conformations further indicated persistent predicted interactions between the ligands and MAO-B active-site residues.

Based on the combined interpretation of docking, ADMET, and molecular-dynamics results, Calothrixin B, Scytonemin, and Zeaxanthin were identified as computationally prioritized candidates, with each compound exhibiting distinct advantages and limitations across the evaluated parameters. Although Scytonemin exhibited the most favorable predicted docking score (−11.9 kcal/mol), Calothrixin B demonstrated the most balanced overall computational profile, particularly owing to its favorable predicted gastrointestinal absorption and blood–brain barrier permeability, together with a comparatively stable ligand trajectory during MD simulation. Scytonemin ranked second, supported by its strongest predicted docking score and lower residue fluctuations in the 460–500 region; however, its highly fluctuating ligand trajectory and unfavorable predicted pharmacokinetic properties reduced its overall computational ranking. Zeaxanthin ranked third because of its comparatively weaker predicted docking score (−11.5 kcal/mol), low predicted gastrointestinal absorption, absence of predicted blood–brain barrier permeability, and relatively higher residue fluctuations around the 460–500 region, despite exhibiting a stable ligand trajectory.

Since MAO-A was not evaluated, MAO-B selectivity cannot be established from the present study and requires further comparative experimental validation. Direct MAO-B enzyme inhibition studies using purified enzyme, including kinetic evaluation under different pH conditions, were not performed, and the proposed antiparkinsonian activity was not experimentally validated in animal models. In addition, validation using a known noncovalent MAO-B inhibitor was not performed. Therefore, the present findings should be considered exploratory computational predictions of potential MAO-B interactions rather than confirmation of MAO-B inhibitory activity. Nevertheless, the integrated computational profiles of Calothrixin B, Scytonemin, and Zeaxanthin identify these compounds as promising candidates for further investigation in the context of MAO-B-targeted drug discovery. In particular, Calothrixin B may represent the most suitable candidate for subsequent experimental evaluation based on its comparatively favorable overall computational profile, while Scytonemin and Zeaxanthin also warrant further investigation to determine their biological relevance.

Future studies should include validation using a known noncovalent MAO-B inhibitor, targeted active-site and/or ensemble docking, purified MAO-B inhibition and kinetic assays, experimental evaluation of pharmacokinetic properties, and appropriate in vitro and in vivo studies. Such investigations will be essential to validate the predicted MAO-B interactions, determine the actual inhibitory potency of these compounds, and establish their potential as lead candidates for the development of novel therapeutic strategies for Parkinson’s disease.

## Figures and Tables

**Table 1:** Docking scores of cyanobacterial bioactive compounds and Safinamide (control) with MAO-B protein.

S. No.	Compound Name	Docking Score (kcal/mol) (MAO-B)
A	Safinamide (control)	−9.5
1	Ambigol A	−7
2	Ambigol B	−7.9
3	Ambiguine H Isonitrile	−7.6
4	Ambiguine I Isonitrile	−7.3
5	Aplysiatoxin	−8.1
6	Astaxanthin	−8
7	Barbamide	−7.4
8	Calothrixin B	−11.7
9	Canthaxanthin	−8.7
10	Carazostatin	−7.4
11	Coibacin A	−8.8
12	Curacin A	−6.5
13	Curacin D	−6.9
14	Debromoaplysiatoxin	−8
15	Echinenone	−10.8
16	Ethyl Tumonoate A	−8.2
17	Fischerellin A	−7.3
18	Fischerindole L	−6.7
19	Gallic acid	−7.2
20	Grenadamide	−6.4
21	Hapalindole A	−7
22	Herbamide B	−7.1
23	Homoanatoxin A	−6.1
24	Honaucin A	−6.6
25	Kalkitoxin	−6.8
26	Luteolin 7-O-glucoside (Cynaroside)	−10
27	Lyngbic aid	−5.7
28	Lyngbyabellin A	−7
29	Lyngbyatoxin A	−7
30	Merocyclophane A	−7.8
31	Mycosporine-2-glycine	−6.2
32	Nocuolin A	−8.1
33	Nostocarboline	−5.5
34	Nostocine A	−5.8
35	Palmyramide A	−7.7
36	Rutin	−8.9
37	Scytonemin	−11.9
38	Scytoscalarol	−7.6
39	Tanikolide	−6
40	Tjipanazole J	−9.8
41	Ulongamide A	−7.6
42	Unnarmicin D	−8.9
43	Venturamide B	−7.5
44	Welwitindolinone A isonitrile	−6.9
45	Zeaxanthin	−11.5

**Table 2:** Molecular docking interactions of Safinamide (control), Calothrixin B, Scytonemin, and Zeaxanthin with the target protein, showing docking scores, interacting amino acid residues, bond categories, interaction types, and interaction distances.

Ligand	Docking Score (Kcal/mol)	Residue in Contact	Bond Category	Interaction Type	Distance (Å)
Safinamide (control)	−9.5	ILE1	Hydrogen Bond	Conventional Hydrogen Bond	3.11289
		SER15	Hydrogen Bond	Conventional Hydrogen Bond	2.85958
		ALA263	Hydrogen Bond	Conventional Hydrogen Bond	2.46231
		VAL10	Hydrogen Bond	Carbon Hydrogen Bond	3.58747
		ILE264	Hydrophobic	Pi-Sigma	3.63391
		TYR393	Hydrophobic	Pi-Pi T-shaped	4.89075
		ALA35	Hydrophobic	Pi-Alkyl	4.3873
Calothrixin B	−11.7	ASN116	Hydrogen Bond	Conventional Hydrogen Bond	2.68509
		THR196	Hydrogen Bond	Carbon Hydrogen Bond	3.48149
		ASP123	Electrostatic	Pi-Anion	3.8321
		GLU483	Electrostatic	Pi-Anion	4.82146
		PHE103	Hydrophobic	Pi-Pi Stacked	4.59095
		TRP119	Hydrophobic	Pi-Pi T-shaped	4.86385
Scytonemin	−11.9	ARG120	Hydrogen Bond	Conventional Hydrogen Bond	3.25762
		ASN116	Hydrogen Bond	Conventional Hydrogen Bond	2.34249
		GLU483	Electrostatic	Pi-Anion	4.85113
		THR196	Hydrogen Bond	Pi-Donor Hydrogen Bond	3.56849
		THR478	Hydrogen Bond	Pi-Donor Hydrogen Bond	3.79306
		PHE103	Hydrophobic	Pi-Pi Stacked	4.81099
		PRO102	Hydrophobic	Pi-Alkyl	5.15371
Zeaxanthin	−11.5	ILE199	Hydrogen Bond	Conventional Hydrogen Bond	2.83587
		GLN206	Hydrogen Bond	Conventional Hydrogen Bond	2.75459
		ARG42	Hydrophobic	Alkyl	4.17888
		PRO265	Hydrophobic	Alkyl	5.05521
		ALA439	Hydrophobic	Alkyl	4.86472
		ILE14	Hydrophobic	Alkyl	3.55656
		MET436	Hydrophobic	Alkyl	5.253
		ARG36	Hydrophobic	Alkyl	4.50234
		TYR398	Hydrophobic	Pi-Alkyl	5.37996

**Table 3:** Physicochemical properties and drug-likeness parameters for the selected cyanobacterial compounds versus the control (Safinamide).

Molecules	Calothrixin B	Scytonemin	Zeaxanthin	Safinamide
**Molecular weight**	298.29 g/mol	544.55 g/mol	568.87 g/mol	302.34 g/mol
**H-bond acceptors**	3	6	2	4
**H-bond donors**	1	4	2	2
**Rotatable bonds**	0	3	10	7
**TPSA Å^2^**	62.82	99.32	40.46	64.35
**Consensus Log(Po/w)**	2.71	5.80	9.83	2.56
**Lipinski: violations**	0	1	2	0
**Bioavailability Score**	0.55	0.55	0.17	0.55

**Table 4:** Pharmacokinetic parameters of the selected compounds.

Compounds	Calothrixin B	Scytonemin	Zeaxanthin
GI absorption	High	Low	Low
BBB permeant	Yes	No	No
P-gp substrate	No	No	Yes
CYP1A2 inhibitor	Active	Inactive	Inactive
CYP2C19 inhibitor	Inactive	Inactive	Inactive
CYP2C9 inhibitor	Active	Active	Active
CYP2D6 inhibitor	Inactive	Inactive	Inactive
Log *K*_p_ (skin permeation)	−2.02 cm/s	−5.45 cm/s	−5.56 cm/s

**Table 5:** Chemical structures, PubChem IDs, and sources of the selected cyanobacterial compounds and the standard drug.

S. No	Name of the Ligand	Source	PubChem ID	Structure
A	Safinamide (control)	-	131682	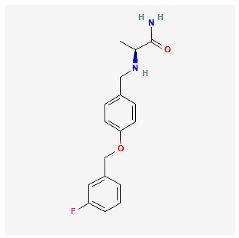
1	Ambigol A	*Fischerella ambigua*	475341	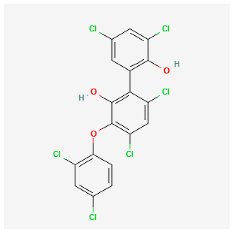
2	Ambigol B	*Fischerella ambigua*	475342	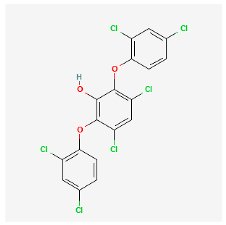
3	Ambiguine H Isonitrile	*Fischerella*	139588184	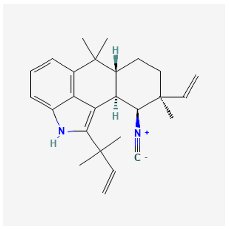
4	Ambiguine I Isonitrile	*Fischerella*	16109784	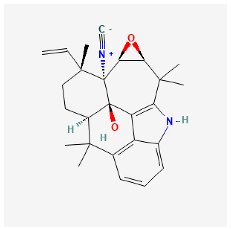
5	Aplysiatoxin	*Lyngbya majuscula*	21672114	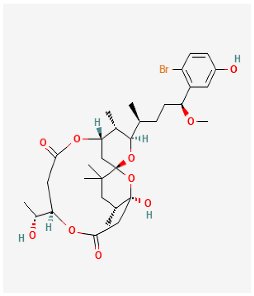
6	Astaxanthin	*Synechocystis* sp. or *Synechococcus elongatus*	5281224	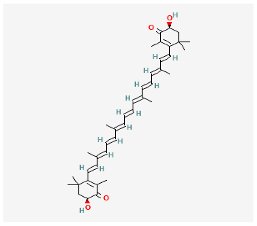
7	Barbamide	*Lyngbya majuscula*	10695334	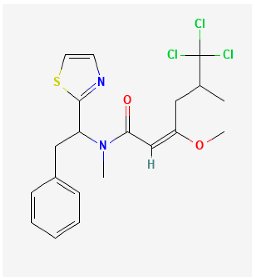
8	Calothrixin B	*Calothrix cyanobacteria*	9817721	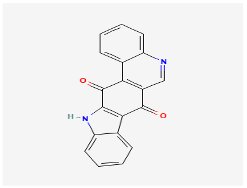
9	Canthaxanthin	*Lyngbya* sp.	5281227	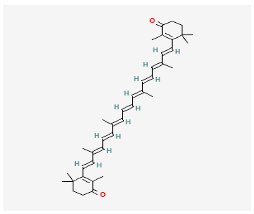
10	Carazostatin	*Hyella caespitose*	130857	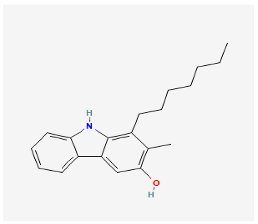
11	Coibacin A	*Oscillatoria* sp.	60203968	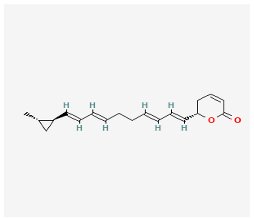
12	Curacin A	*Lyngbya majuscule*	5281967	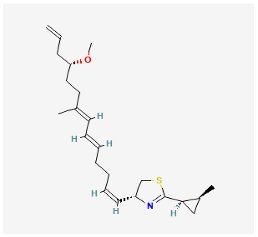
13	Curacin D	*Lyngbya* sp.	10546404	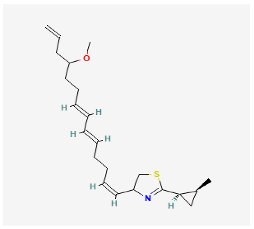
14	Debromoaplysiatoxin	*Lyngbya majuscula*	5352033	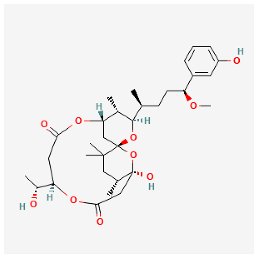
15	Echinenone	*Planktothrix rubescens*, *Planktothrix agardhii* *Tychonema* sp.	5281236	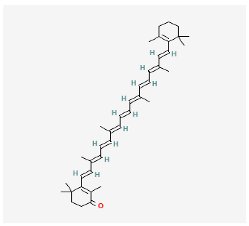
16	Ethyl Tumonoate A	*Oscillatoria margaritifera*	53493313	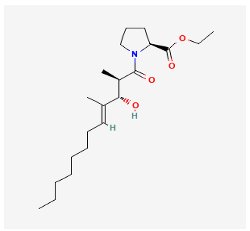
17	Fischerellin A	*Fischerella muscicola*	139587195	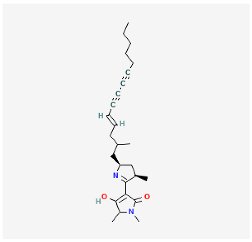
18	Fischerindole L	*Fischerella muscicola*	10065777	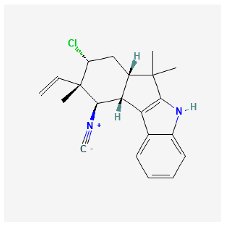
19	Gallic acid	*A. doliolum*, *O. acuta*, *P. boryanum*, and *H. intricatus*	370	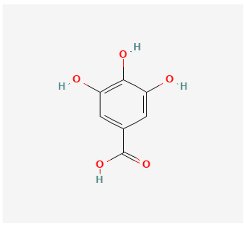
20	Grenadamide	*Lyngbya majuscula*	10710472	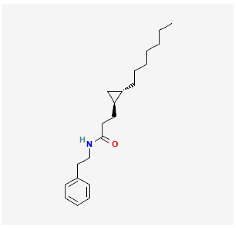
21	Hapalindole A	*Fischerella muscicola*	185159	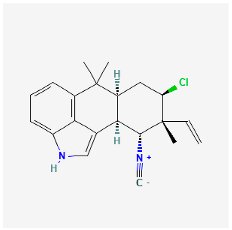
22	Herbamide B	*Lyngbya majuscula*	44627702	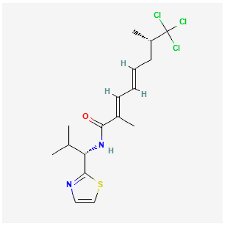
23	Homoanatoxin A	*Oscillatoria PCC*	171380042	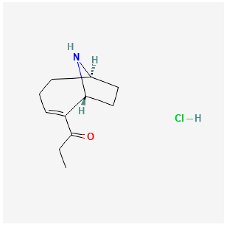
24	Honaucin A	*Leptolyngbya crosbyana* strain	54759250	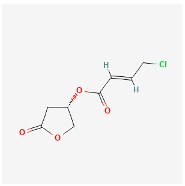
25	Kalkitoxin	*Lyngbya majuscula*	11176051	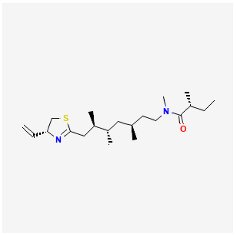
26	Luteolin 7-O-glucoside (Cynaroside)	*Leptolyngbyba* sp.	5280637	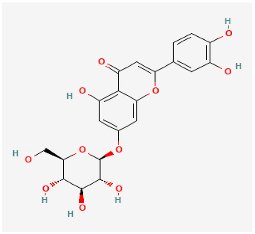
27	Lyngbic acid	*Lyngbya majuscula*	11065049	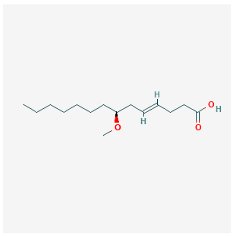
28	Lyngbyabellin A	*Lyngbya majuscula*	10032587	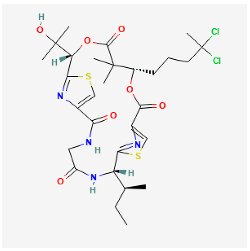
29	Lyngbyatoxin A	*Lyngbya majuscula*	91706	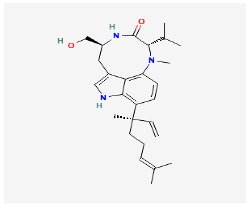
30	Merocyclophane A	*Nostoc* sp.	62706357	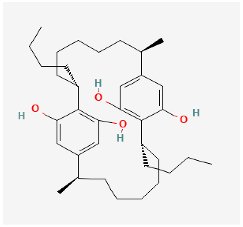
31	Mycosporine-2-glycine	*Aphanothece halophytica*	23427657	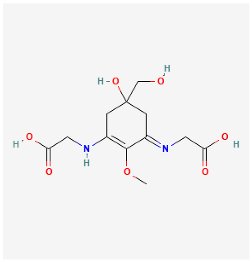
32	Nocuolin A	*Nostoc* sp.	129017397	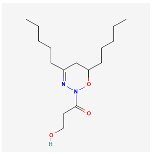
33	Nostocarboline	*Nostoc* sp.	5326150	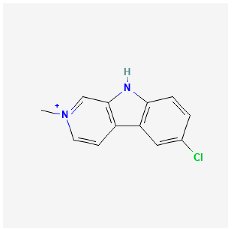
34	Nostocine A	*Nostoc* sp.	10749358	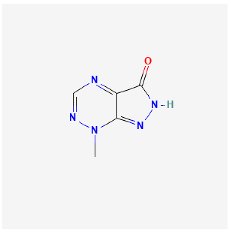
35	Palmyramide A	*Oscillatoria* spp.	45380200	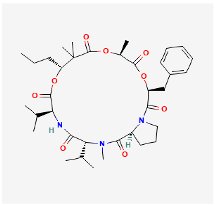
36	Rutin	*Microcheate tenera*	5280805	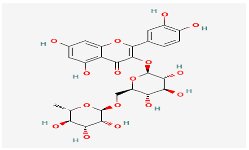
37	Scytonemin	*Scytonema* sp.	135473381	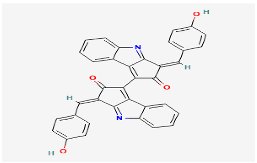
38	Scytoscalarol	*Scytonema* sp.	44605340	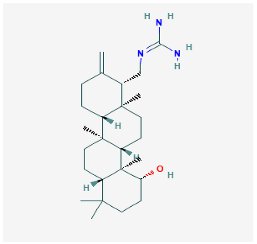
39	Tanikolide	*Lyngbya majuscula*	5276592	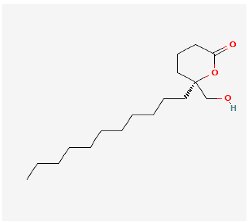
40	Tjipanazole J	*Tolypothrix tjipanasensis*	146683633	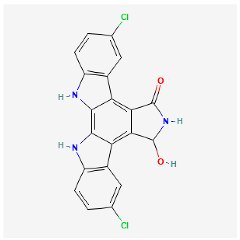
41	Ulongamide A	*Lyngbya* sp.	10985040	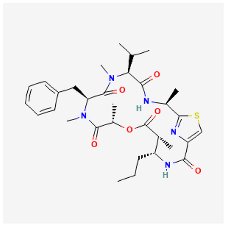
42	Unnarmicin D	*Trichodesmium thiebautii*	139590496	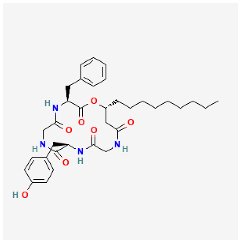
43	Venturamide B	*Oscillatoria* sp.	16115401	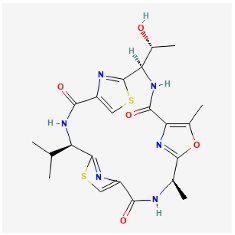
44	Welwitindolinone A isonitrile	*Fischerella muscicola*	11501239	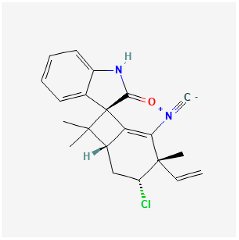
45	Zeaxanthin	*Cyanobium* sp.	5280899	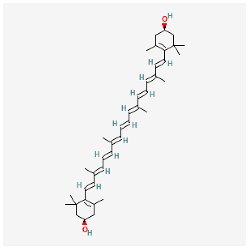

## Data Availability

Data is contained within the manuscript and the Supplementary Materials.

## Supplementary Materials

The following supporting information can be downloaded at: https://www.mdpi.com/article/10.3390/ph19101569/s1, Supplementary File S1: Toxicity prediction report of the investigated compounds, including the predicted toxicity endpoints and related toxicological parameters; Supplementary File S2: SwissADME analysis of the investigated compounds, including physicochemical properties, lipophilicity, water solubility, pharmacokinetic properties, drug-likeness, and medicinal chemistry parameters; Supplementary File S3: Molecular docking results showing the binding energies of the investigated compounds across independent docking runs.

## Abbreviations

The following abbreviations are used in this manuscript:

RMSD	root mean square deviation
RMSF	root mean square fluctuation
ADMET	absorption distribution metabolism excretion and toxicity
MD	molecular dynamics
PDB	Protein Data Bank
